# Defense Mechanisms and Treatment Response in Depressed Inpatients

**DOI:** 10.3389/fpsyg.2021.633939

**Published:** 2021-03-18

**Authors:** Yves de Roten, Slimane Djillali, Fabienne Crettaz von Roten, Jean-Nicolas Despland, Gilles Ambresin

**Affiliations:** ^1^Institute of Psychotherapy, Department of Psychiatry, University Hospital Center and University of Lausanne, Lausanne, Switzerland; ^2^Faculty of the Social and Political Sciences, University of Lausanne, Lausanne, Switzerland

**Keywords:** defense, depression, treatment response, inpatient, psychodynamic psychotherapy, brief psychotherapy, psychotic defense

## Abstract

The study investigated the extent to which defensive functioning and defense mechanisms predict clinically meaningful symptomatic improvement within brief psychodynamic psychotherapy for recurrent and chronic depression in an inpatient setting. Treatment response was defined as a reduction in symptom severity of 46% or higher from the baseline score on the Montgomery–Asberg Depression Rating Scale (MADRS). A subsample of 41 patients (19 responders and 22 non-responders) from an RCT was included. For each case, two sessions (the second and the penultimate) of brief inpatient psychodynamic psychotherapy (a manualized 12-session therapy program developed in Lausanne) were transcribed and then coded using the Defense Mechanism Rating Scales (DMRS) and the Psychotic Defense Mechanism Rating Scales (P-DMRS), an additional scale developed to study psychotic defenses. Results showed that defensive functioning and mature and immature defense changed during psychotherapy and predicted treatment response. Patient’s defenses observed throughout therapy also predicted treatment response at 12-month follow-up. The addition of psychotic defenses allows a better prediction of the treatment response. Overall, these results are in line with previous research and provide further validation of defensive functioning as a predictor of outcomes and a mechanism of change in psychotherapy.

## Introduction

From an empirical perspective, psychological defenses might be viewed either as a patient trait that determines the course and outcome of treatment, as a therapeutic outcome that evolves toward more adaptability, or as an underlying mechanism of change that explains how psychotherapy works from the psychodynamic theoretical perspective.

Studies have suggested that defenses can be associated with depression. Compared to a healthy control group, depressed individuals were found to use significantly more maladaptive and fewer adaptive defense mechanisms at baseline (Vaillant, 1986). DeFife and Hilsenroth (2005) showed that the presence and severity of depression symptoms were significantly related to lower (more maladaptive) overall defensive functioning (ODF) scores. In addition, patients who lack obsessional defenses of mental inhibition (including isolation, undoing, and intellectualization) are more severely depressed. Compared to panic disorder, Calati et al.’s (2010) meta-analysis confirmed a specific defensive profile related to depression, characterized by a low level of mature and a high level of immature defenses.

A group of eight immature defenses, called depressive defenses (help-rejecting complaining, acting out, splitting of self-image, splitting of others’ images, projective identification, devaluation of self-image, devaluation of others’ images, and projection), hypothesized to play a causal role in depression, were found to predict the course of major depression in a sample of psychiatric patients. Six months after intake, immature defenses were identified more often in depressed patients who improved less than predicted by their initial functional status and one high adaptive level defense (self-observation) was identified more often in those who improved more than predicted by their initial status (Hoglend and Perry, 1998). These depressive defenses were associated with lower patient improvement on global functioning 6 months after intake (Hoglend and Perry, 1998). The group of other immature defenses (so-called non-depressive immature defenses) was not related to improvement. This confirmed the results of Bloch et al. (1993), who found that these defenses occurred more frequently in a sample of dysthymic patients compared to patients with panic disorder. Compared to patients with anxiety disorders, outpatients diagnosed with depression had significantly lower ODF and a higher proportion of maladaptive defenses at the beginning of treatment. However, depressed patients responded better to treatment, with higher increase in ODF than patients with anxiety disorders had (Babl et al., 2019).

In studies involving primarily depressed patients, defensive functioning improved with an increase in ODF during therapy. This outcome relates to a specific pattern of defense mechanism evolution, whereby the proportion of high adaptive defenses increases, and the proportion of maladaptive defenses decreases (Kneepkens and Oakley, 1996; Akkerman et al., 1999; Drapeau et al., 2003; Bond and Perry, 2004; Perry and Bond, 2009; Kramer et al., 2013; Babl et al., 2019), more specifically depressive defenses (DeFife and Hilsenroth, 2005; Perry et al., 2020). With other disorders, ODF also significantly increases but alongside other patterns of defense mechanism evolution (Perry and Bond, 2017). As suggested by Cramer’s (1998) review, these results should be seen from the vantage point of adaptational processes that serve an individual’s need for adaptation; defenses may be understood as an individual’s way of responding to their need to adapt. In a sample of patients with personality disorders, although some individuals improved significantly after 1 year of therapy, the group did not show significant change in defenses (Perry, 2001). Longer-term treatments are commonly required to effect significant improvement in defensive functioning. Perry and Bond (2009) provided preliminary evidence on change in ODF over 2.5 years of therapy for three cases with different personality disorder types.

Relatively few studies have directly examined the extent to which defensive functioning and defense mechanisms predict outcomes in depressive disorders. In a pilot study of 12 patients with recurrent major depression, Perry et al. (2020) showed that the mean percentage score of depressive defenses significantly decreased after 20 sessions of psychotherapy (mean ES = 0.97) and improved defensive functioning led to overall mental health improvement. However, patients had not maintained this result after 12 months of follow-up. In a sample of young adults with adjustment disorders (mainly with mixed anxiety and depressive symptoms), Kramer (2010) showed prior improvement in defensive functioning mediated change in distress. The short-term mutability of mature and immature defenses was also found in cluster C personality disorders treated with cognitive behavioral therapy (Johansen et al., 2011).

In long-term psychodynamic psychotherapy of a heterogeneous sample of patients with anxiety, depression, and personality disorders, Bond and Perry (2004) reported that defenses accounted for larger outcome-variance change than initial symptomatic severity did. Moreover, improvement in ODF score predicted improvement in observer-rated depression, even after controlling for improvement in distress. A more mature defensive functioning was highly associated with improvement in symptom levels and functioning 5 years after intake (Perry and Bond, 2012). However, these studies considered the outcome solely from a statistical point of view, which means that they sought to disprove a negative and state an event probably did not happen by chance. By contrast, clinical significance seeks to prove a positive, and state an event genuinely happened (de Roten and Crettaz von Roten, 2018). Reliance on statistical change does not directly address whether subjects improved clinically or recovered.

This paper explores the extent to which defensive functioning and defense mechanisms predict improvement and recovery in short-term dynamic psychotherapy for recurrent or chronic depression. We address whether (a) defensive functioning and defense mechanisms help improve adaptiveness or maturity with therapy, (b) defenses and change in defenses are associated with treatment response and remission, and (c) defenses and change in defenses are associated with maintenance of treatment response after 12 months of follow-up.

## Materials and Methods

### Sample

A subsample was selected from a randomized controlled trial on the efficacy of adjunctive brief psychodynamic psychotherapy in usual inpatient treatment of depression (de Roten et al., 2017). For more detail on the design of the study, see Ambresin et al. (2012). From among the 76 patients in the psychotherapy group of the main study, 41 were included. Selection criteria required patients to have completed at least 10 sessions (*n* = 52), including two sessions (the second and the penultimate) that were audio or video recorded. Univariate tests showed that the subsample’s demographics and clinical variables were not different from those of the whole sample (see Supplementary Table 1).

To be included in the main study, patients hospitalized in the university psychiatric hospital had to (a) meet *Diagnostic and Statistical Manual of Mental Disorders IV* criteria for unipolar major depressive episode; (b) be aged 18–65 years, (c) have a Montgomery–Asberg Depression Rating Scale (MADRS; Montgomery and Asberg, 1979) score > 18, and (d) have sufficient command of French. Exclusion criteria were limited to bipolar disorders, psychotic disorder, and persistent substance use/abuse that might affect brain function (memory, level of consciousness, and cognitive abilities) and impair an individual from participating in and benefiting from psychotherapy.

Table 1 shows demographic and clinical characteristics of the responders (*n* = 19) and non-responders (*n* = 22). Univariate comparisons showed no differences. The 16 therapists (10 women and six men) who participated had completed (10 senior therapists) or were in the advanced stage of completing (six junior therapists) 5 years of psychotherapy training. The junior and senior therapists had no differences in their patients’ response and remission rates. They attended a weekly training seminar dedicated to inpatient brief psychodynamic psychotherapy (IBPP) for 6 months before they started their first IBPP sessions with a patient. We monitored for adherence and competence through weekly individual supervision and continued participation in the training seminar.

**TABLE 1:** Demographic and clinical characteristics of the sample.

Variable	Responders (*n* = 19)	Non-responders (*n* = 22)	*p*
Age	42.8 (9.5)	45.5 (9.8)	0.393
Gender (female)	13 (68.4%)	14 (63.6%)	0.829
Education (years)	11.0 (3.4)	9.3 (2.3)	0.064
**Marital status**			
Single	3 (15.8%)	4 (18.1%)	
Couple	8 (42.1%)	10 (45.5%)	
Divorced/widowed	8 (42.1%)	8 (36.4%)	0.877
Chronicity	9 (47.4%)	12 (54.5%)	0.752
Tentamen	11 (57.9%)	8 (36.4%)	0.739
Early onset	6 (31.6%)	7 (37.8%)	0.631
Duration of current episode	82.5 (101.0)	71.1 (79.7)	0.747
Childhood trauma (CTQ)	2.3 (0.4)	2.3 (1.1)	0.962
Length of hospital stay	42.5 (38.1)	45.2 (42.9)	0.829

*Statistical tests were *t*-test or Fisher exact test; CTQ, Childhood Trauma Questionnaire.*

### Instruments

#### Outcome

We used the MADRS, a clinician-rating measure that uses 10 items to provide a sensitive measure of patient change in inpatient settings. Davidson et al. (1986) demonstrated the construct validity of MADRS using an inpatient sample.

Research psychologists (master’s level), who were not involved in the inpatient care and not located in the hospital, administered the MADRS. Inter-rater reliability was obtained from 15 audiotaped interviews, mean *ICC*(2,1) = 0.88, range = 0.68–0.96. In our study, Cronbach’s *α* = 0.85. Response and remission were suggested as the most relevant outcome criteria for the treatment of depression. In line with Riedel et al. (2010), we defined response *a priori* as a reduction in symptom severity of 46% or higher from the baseline score and remission as a score of 7 or less, based on cut-off scores determined in a large inpatient population. Nineteen patients (46.3%) responded positively to treatment.

We also used the Quick Inventory of Depressive Symptomatology (QIDS-SR_16_; Corruble et al., 1999), a 16-item self-report measure, to evaluate depressive symptomatology. In our study, Cronbach’s *α* = 0.81. Response corresponded to a symptom reduction ≥ 50%, and remission to a score of score of 5 or less, according to Rush et al. (2006). Supplementary Table 2 provides the results for this instrument. Nineteen cases were responders, according to the QIDS-SR_16_. Thirteen cases (68.4% of the responders) were responders, according to both MADRS and QIDS, and six cases were responders in only one measure. Three cases were responders according to MADRS but not according to QIDS, and three cases were responders according to QIDS but not according to MADRS.

#### Defense Mechanisms

The Defense Mechanism Rating Scales (DMRS; Perry et al., 2004) is an observer-based method that identifies any of 30 individual defense mechanisms as they occur in verbatim transcripts of therapy sessions or interviews. These mechanisms are hierarchically arranged in seven defense levels (1–7) according to their adaptiveness, from the least adaptive to the most adaptive: action, major image distorting, disavowal, minor image distorting, neurotic, obsessional, and high adaptive. We added the Psychotic-Level Defense Mechanisms Rating Scale (P-DMRS; Berney et al., 2014). This eighth defense level includes six psychotic defense mechanisms (psychotic denial, autistic withdrawal, distortion, delusional projection, fragmentation, and concretization). As is the case in the DMRS, each defense of the P-DMRS is extensively described (i.e., definition, function, discrimination, and rating) in a manual (Berney et al., 2010). A detailed presentation of the P-DMRS with clinical examples for each defense mechanism can be found in Berney et al. (2014). The first validation was conducted on a sample of 80 patients with depressive disorder (*n* = 20), bipolar disorder (*n* = 20), personality disorder (*n* = 20), and schizophrenic disorder (*n* = 20). The validation showed that (a) psychotic defenses can be reliably identified in transcripts of psychotherapy sessions, (b) psychotic defenses can be present in a wide range of defensive functioning, and (c) the new scale has psychometric characteristics similar to those of the other subscales of the DMRS (Berney et al., 2011).

Combinations of defense level scores define mature (high adaptive defenses, including affiliation, altruism, anticipation, humor, self-assertion, self-observation, sublimation, and suppression), intermediate (obsessional and neurotic defenses), and immature defense category (psychotic, action, major image distorting, and disavowal defenses). Moreover, the depressive defense category is comprised of eight immature defenses (passive–aggressive, acting out, help-rejecting complaining, projective identification, splitting of self-images, splitting of others’ images, projection, and devaluation) empirically associated with depression, whereas non-depressive defenses are comprised of autistic fantasy, rationalization, denial, idealization, and omnipotence (Hoglend and Perry, 1998).

Scores represent the relative frequency per defense level and defense category, culminating in a weighted score, referred to as the ODF score—of the relative frequencies of all defense mechanisms by their level. Level zero has been attributed to psychotic defenses, making the ODF score comparable to other studies without considering psychotic level. For the current study, reliability coefficients on 18 transcripts (22% of the ratings) were established among four trained raters and yielded satisfactory results at the level of defense, with *ICC*(2,1) varying between 0.69 and 0.94 (*M* = 0.77; *SD* = 0.12) for the early session and between 0.71 and 0.95 (*M* = 0.78; *SD* = 0.11) for the late session. At the level of ODF score, *ICC*(2,1) were higher, with a mean of 0.85 (SD = 0.09).

### Treatment

The IBPP is a manualized 12-session psychodynamic psychotherapy program developed in Lausanne. IBPP is based on the *Psychodynamic Treatment of Depression* manual developed by Busch et al. (2004) to help therapists focalize on relevant depression topics, as well as on the *Brief Psychodynamic Psychotherapy* manual developed by Despland et al. (2010) for work on transference, personality organization, and conflictual themes. The initial hypothesis was based on the dynamic relationship established between a therapist and a patient during the first three sessions (pre-transference), a patient’s present crisis, and the dynamics that form the core of a patient’s depressive episode. Subsequent sessions focus on helping the patient gain a fuller understanding of the psychological factors that led to the emergence of depressive symptoms and address their vulnerability to those dynamics. Final sessions address the patient’s feelings and fantasies about termination, as well as the decision regarding a longer term therapy or ongoing psychiatric treatment if necessary. Treatment integrity was checked (de Roten et al., 2017).

### Procedure and Analysis

All psychotherapy sessions were audio- or videotaped. From each case, two sessions (the second and the penultimate) were transcribed, according to the method defined by Mergenthaler and Stigler (1997). Five fully trained raters carried out DMRS and P-DMRS ratings. The first author provided initial weekly group-training sessions that lasted 12 weeks and subsequent calibration of raters over 3 months.

Analysis was performed using SPSS version 26. Effect sizes were within-condition, taking the correlation between the pre- and posttest into account (Morris and DeShon, 2002). We used linear mixed models (LMM) to study the effect of time, treatment response, and the interaction (Time × Response), which were treated as fixed effects for each defense category and each defense level, with the MADRS score at intake as covariate. Linear regressions were used to evaluate the relation between defenses and MADRS at the same session, using forward stepwise selection due to sample size.

## Results

### Do Defenses Evolve During the Therapy?

Table 2 shows changes in defensive functioning, defense categories, and defense levels after the psychotherapy. ODF increased throughout therapy, yielding a large effect size. Changes in the mature defense category indicated an increase of mature defensive behaviors in therapy sessions by the end of therapy, as indicated by a moderate positive effect size. Immature and depressive defense categories decreased, as shown by moderate negative effect sizes. Concerning defense levels, the high adaptive level increased in frequency, as indicated by a moderate positive effect size.

**TABLE 2:** Change in defenses.

Defenses	*d*	95% CI	
		LL	UL
Overall defensive functioning	0.727	0.348	1.248
**Defense categories**			
Mature	0.510	0.147	1.031
Intermediate	0.405	−0.016	0.859
Immature	−0.543	−1.017	−0.134
Depressive	−0.559	−1.015	−0.131
**Defense levels**			
High adaptive	0.510	0.147	1.031
Obsessional	0.382	−0.010	0.865
Neurotic	0.116	−0.313	0.533
Minor image-distorting	−0.340	−0.803	0.070
Disavowal	−0.240	−0.652	0.216
Major image-distorting	−0.417	−0.783	0.089
Action	−0.146	−0.586	0.281
Psychotic	0.055	−0.381	0.485

*Immature category, psychotic + action + major image-distorting + disavowal levels; CI, confidence interval; LL, lower limit; UL, upper limit.*

### Do Defenses Predict Response and Remission?

We first looked at the relation between defenses and depression in the same session. Depressive symptom severity was not significantly correlated with depressive defenses (*r* = 0.083 for the second session and 0.255 for the penultimate session). For the eight defensive levels, a forward stepwise regression showed, for the second session, an adjusted *R*^2^ of 0.277, and three defensive levels included in the final step (obsessional, narcissistic, and major image distorting), all significant (*p* = 0.005, 0.011, and 0.011, respectively). For the penultimate session, the adjusted *R*^2^ was 0.092, with only action level included (*p* = 0.030).

**FIGURE 1:**
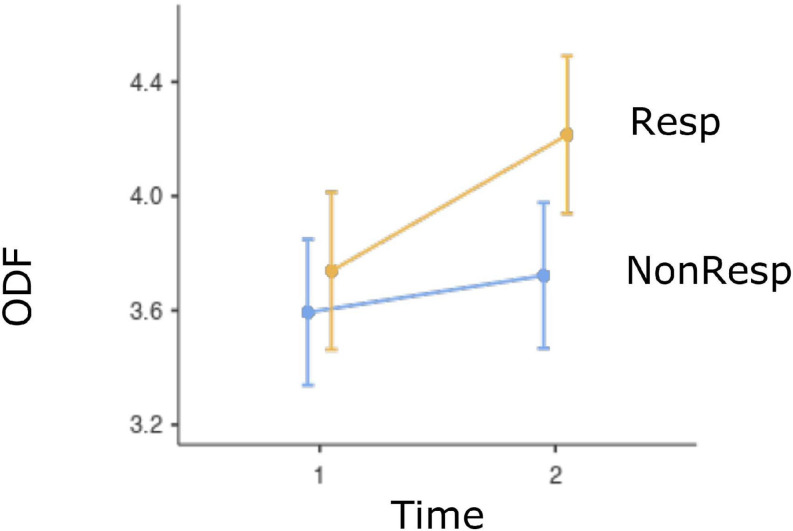
Fixed effect plot with 95% CI for the evolution of ODF for responders and non-responders. *Note.* ODF, overall defensive functioning; Resp, responders; Non-Resp, non-responders; Time 1, pretherapy; Time 2, posttherapy.

**FIGURE 2:**
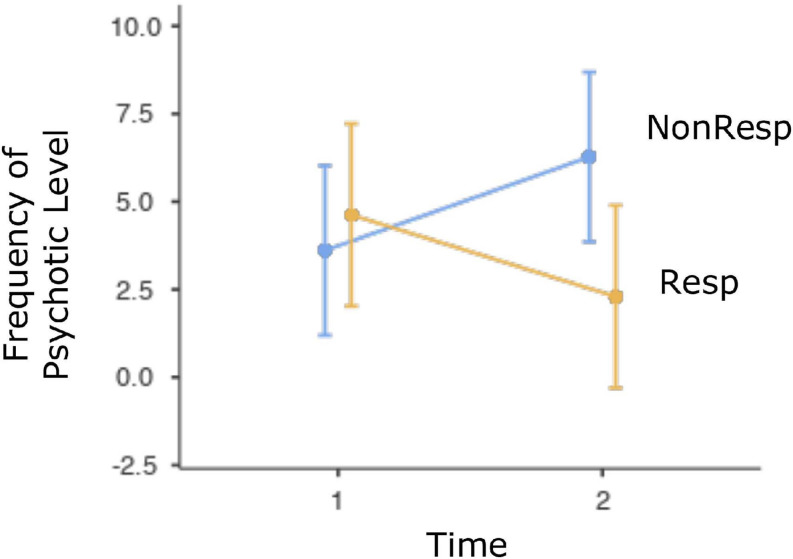
Fixed effect plot with 95% CI for the evolution of psychotic level for responders and non-responders. *Note.* Resp, responders; Non-Resp, non-responders; Time 1, pretherapy; Time 2, posttherapy.

Relation between defenses and treatment response and remission is presented in Table 3. Only defense categories and defense level with significant results are displayed. LMM provided strong evidence that high adaptive and psychotic defense levels were associated with the interaction between time and response, whereas moderate evidence was found for an association among ODF, immature defense category, and the interaction between time and response. Strong evidence for an association among ODF, high adaptive level, and response was found, whereas moderate evidence indicated an association between the immature category, psychotic level, and response. Intermediate and depressive defense categories, as well as neurotic, minor image-distorting, disavowal, and major image-distorting defense levels were not significantly related to response or the interaction between time and response. Figure 1 illustrates the interaction between response and time by displaying the increase in ODF associated with response. Figure 2 displays the decrease in frequency of psychotic defenses associated with response.

**TABLE 3:** Relation among defenses and treatment response and remission.

	Response					Remission		
Defenses	Estimate	*p*	95% CI		Estimate	*p*	95% CI	
			LL	UL			LL	UL
**Overall defensive functioning**								
Time	−0.476	0.000	−0.677	−0.275	−0.457	0.005	−0.765	−0.149
Response	−0.489	0.009	−0.852	−0.126	−0.222	0.371	−0.715	0.271
Time × Response	0.348	0.014	0.073	0.622	0.214	0.222	−0.134	0.562
**Mature category/high adaptive level**								
Time	−6.521	0.000	−9.165	−3.877	−4.322	0.048	−8.601	−0.043
Response	−4.148	0.007	−7.138	−1.159	2.181	0.267	−1.706	6.069
Time × Response	5.857	0.002	2.248	9.467	1.210	0.616	−3.634	6.054
**Psychotic level**								
Time	2.326	0.078	−0.273	4.926	2.067	0.306	−1.960	6.093
Response	4.073	0.026	0.513	7.632	2.603	0.271	−2.087	7.292
Time × Response	−4.990	0.007	−8.539	−1.441	−3.098	0.177	−7.656	1.460
**Immature category**								
Time	−10.395	0.000	−15.127	−5.662	9.567	0.005	3.141	15.993
Response	−9.415	0.020	−17.283	−1.547	4.769	0.347	−5.329	14.866
Time × Response	6.545	0.047	0.084	13.005	−5.229	0.154	−12.503	2.045

*Immature defenses include psychotic, action, borderline, and disavowal levels; CI, confidence interval; LL, lower limit; UL, upper limit.*

Nine cases (22.0%) were remitted at the end of the psychotherapy. No defense category or defense level showed significant results in terms of treatment response or the interaction of time and response (Time × Response).

### Do Defenses Predict Response and Remission at Follow-Up?

Sixteen cases (39.0%) were responders at 12-month follow-up. Thirteen cases were also remitters at the end of the psychotherapy. All significant results for defense categories and levels are presented in Table 4. Very strong evidence for an association among ODF, mature defense category, and response was found, whereas moderate evidence for an association between the immature defense category and response was found. Regarding the interaction between time and response, very strong evidence was found for an association with the mature defense category, whereas strong evidence for an association with ODF was found. The immature defense category was not significantly associated with the interaction of time and response.

**TABLE 4:** Relation among defenses and treatment response and remission after 12-month follow-up.

	Response					Remission		
Defenses	Estimate	*p*	95% CI		Estimate	*p*	95% CI	
			LL	UL			LL	UL
**Overall defensive functioning**								
Time	−0.473	0.001	−0.690	−0.255	−0.499	0.001	−0.779	−0.219
Response	−0.627	0.000	−0.995	−0.259	−0.645	0.005	−1.080	−0.209
Time × Response	0.403	0.008	0.111	−0.694	0.381	0.027	0.046	0.716
**Mature category/high adaptive level**								
Time	−7.475	0.000	−10.152	−4.798	−7.500	0.000	−11.279	−3.721
Response	−6.142	0.000	−9.120	−3.164	−7.162	0.000	−10.610	−3.716
Time × Response	7.925	0.000	4.333	11.517	6.722	0.005	2.196	11.248
**Psychotic level**								
Time	0.888	0.534	−1.984	3.759	2.310	0.191	−1.210	5.830
Response	4.095	0.022	0.623	7.568	4.099	0.051	−0.022	8.222
Time × Response	−3.253	0.095	−7.105	0.560	−5.188	0.018	−9.405	−0.972
**Immature category**								
Time	6.913	0.007	2.033	11.792	6.610	0.044	0.179	13.041
Response	10.237	0.014	2.169	18.306	11.978	0.010	2.969	20.987
Time × Response	−3.398	0.299	−9.944	3.149	−2.014	0.598	−9.717	5.689

*Immature defenses include psychotic, action, borderline, and disavowal levels; CI, confidence interval; LL, lower limit; UL, upper limit.*

At 12-month follow-up, 30.3% (10 out of 33) of patients were remitted (eight missing values). Only four cases were also remitters at the end of the treatment.

## Discussion

In line with previous research, our study showed that defensive functioning and high adaptive defenses significantly increased, while immature defenses decreased over the course of psychotherapy. Moreover, within the immature defense category, the group of depressive defenses changed the most (*d* = 0.56), which further validates defense mechanisms as relevant and specific mechanisms of change in psychotherapy for depression. However, the effect sizes are lower than those that Perry et al. (2020) found in a sample of patients with recurrent major depression. Contrary to what was expected, the depressive defenses did not predict outcomes.

The original contribution of this study concerned the clinical significance of defenses as predictors of outcomes by examining how defenses predict treatment response and remission. Results showed that the categories of mature and immature defenses predict responses at the end of the treatment and at 12-month follow-up. The most adaptive levels of defense (high adaptive and obsessional) and maladaptive (psychotic and action) levels of defense are related to response and/or interaction between time and response, as seen at the end of the treatment but not at follow-up. Finally, defensive functioning and defense mechanisms are not predictors of remission. These results confirm previous research findings and extend them to the specific context of very brief dynamic psychotherapy for recurrently and chronically depressed inpatients. A great need exists to identify predictors of treatment response, and these results clearly showed that defense mechanisms represent a promising approach.

At the end of the treatment, defenses predicted response but not remission, whereas a stronger effect would have been expected. This may be due to a lack of statistical power. The rate of remission was relatively low: 24% at the end of the therapy and 25% after 12-month follow-up. An alternative explanation is that the remission rate may be related to the duration and goal of the treatment. IBPP is only intended as a first step, which may work as an initial insight facilitating a longer course of psychotherapy after hospitalization. Within the IBPP, remission was not a therapeutic objective and mainly extratherapeutic reasons facilitated it. Results at 12-month follow-up tend to confirm this hypothesis. After discharge from the hospital, 95% of patients included in this study were in psychotherapy. Depressive defenses evolved the most during therapy, but they did not predict response. Thus, change in depressive defenses cannot be considered as an outcome measure. Drapeau et al. (2003) showed that changes in defense mechanisms in very short interventions are likely related to clinical processes reflecting a state-dependent improvement. Some studies showed change in defenses not only in psychodynamic psychotherapy but also in cognitive behavioral therapy (Babl et al., 2019; Perry et al., 2020). These depressive defenses are particularly sensitive to therapeutic work done during hospitalization, and therapeutic progress stems from the goal of the psychotherapy and hospitalization, which is to reduce patients’ acute states of distress. These defenses are particularly strained because of the problems these patients have with recurrent or chronic depression. We may hypothesize that change in these defenses as a trait change occurs only after long-term psychotherapy and the acquisition of adaptive skills (Johansen et al., 2011), as seen with personality disorders Perry and Bond (2009). Contrary to previous studies, these defenses were not associated with depression in our sample. This may be due to the clinical characteristics of our inpatient sample such as treatment resistance, chronicity, and high comorbidity. Compared to Perry et al.’s (2020) study on outpatients with recurrent depression, the proportion of depressive defenses was higher in our sample at intake (17.1 vs 26.8) and after the psychotherapy (8.5 vs 20.1).

In line with our previous work (Berney et al., 2011), psychotic level showed the same inter-rater reliability as the other defense levels of the DMRS. Measuring psychotic defenses proved a useful supplementary approach to examining changes in defenses over the course of psychoanalytic therapies. In our study, psychotic defense level is the most predictive level of maladaptive defense related to outcomes. Moreover, if a patient’s psychological defenses do not evolve during treatment, then an interaction effect between time and response may occur. Successful therapy implies a reduction of these defenses from 5 to 2.5%.

Psychotic defenses were present in 54% of the patients. Among these patients, an examination of their verbatim statements made it possible to study the context in which psychotic defenses appeared in our sample. We found that they tend to appear when the therapeutic interaction is difficult (e.g., when the therapeutic alliance is strained or when the patient is in crisis for an extratherapeutic reason). Further research should explore how patients improve or worsen these defenses seem to be particularly sensitive to how therapy evolves. Inclusion of psychotic defenses in the DMRS provides a better account of patients’ defensive functioning, psychopathologically more valid defensive scores, and a more complete and valid measure of patients’ progress through the course of treatment. From a clinical point of view, training clinicians to detect psychotic defenses as early as possible seems to be important to being responsive to patients’ levels of functioning.

In our sample, inpatients did not present psychotic symptoms that would be coded in phenomenological psychiatric diagnoses. Use of psychotic defenses does not imply the presence of psychotic symptoms (Berney et al., 2014). Inpatients in our sample used unconscious psychotic defense mechanisms to mediate their reaction to emotional conflicts arising from internal and external stressors. Our results suggest that psychotic defense mechanisms is important to consider when studying severe depression in an inpatient setting, alongside its importance in the study of severe personality, bipolar, paranoiac, and schizophrenic disorders. Measuring psychotic defense level may capture psychotic psychological functioning in severely depressed inpatients presenting with extreme features of depression reminiscent of the clinical condition formerly known as *melancholia*. Patients often intertwine and mobilize individual psychotic defense mechanisms together. These mechanisms are difficult to disentangle and often appear in narratives to various degrees in narratives (see Berney et al., 2009). The P-DMRS is comprised of six psychotic defense mechanisms: psychotic denial, autistic withdrawal, distortion, delusional projection, fragmentation, and concretization. Although our results provided evidence that supports the measurement of psychotic level as a whole, below, we provide examples of a few individual psychotic defense mechanisms to illustrate their function in session conditions.

One particular difficulty in treating patients with severe chronic depression is approaching psychic pain and helping them face the unbearable thoughts that often underlie depression (Leuzinger-Bohleber et al., 2019). Clinicians turning their attention to this psychological pain certainly help patients strengthen the self and regain a sense of self-agency. However, therapists can be tempted to avoid addressing defense mechanisms, thereby resounding with their patients’ defense mechanisms. Defending themselves against the reviviscence of traumatic internal or external experiences, severely depressed inpatients can turn mute, cutting themselves off from the distressing reviviscence that the clinical encounter elicits (autistic withdrawal). One of the participants reported: “*I stand in calmness, I shut myself, I close the blinds, I’m in the dark and then, that’s it, I’m fine like this, in the dark. Lying down, I can spend a fortnight like that in my room.*” This excerpt appeared in the penultimate session when the therapist and the patient were exploring the patient’s difficulty coming to therapy: “*I did not want to leave my house, I wanted to stay locked inside the house*….” Therapy was close to its end, and the therapist and the patient were soon to part. The session’s rhythm was slow, and the patient emitted heavy sighs. The narrative of the session started with the patient’s daughter entering a foster home and the difficulty of being separated from her. The patient could hardly speak, and the therapist uttered the following words, adopting the patient’s behavior: “*To do nothing, to avoid any tension inside*… *I prefer to lie down calmly to avoid any tension and pain*… *to be free of conflicts*….” Finally, the patient agreed: “*Yes, I*… *I act like this.*” The excerpt above suggests listening to and working through the psychic pain helped the patient to overcome the flood of painful parting sensation leading to autistic withdrawal, helping her access representations of the blunt pain that had hitherto been indescribable.

Session narratives also suggest that severely depressed inpatients mobilized distortion, understood as a gross altering or reshaping of internal or external reality. Inpatients may modify the representation of reality in a depressive way, reminding us of Freud’s (1917) “Mourning and Melancholia” (p. 245): “The [melancholic] patient represents his ego to us as worthless, incapable of any achievement and morally despicable: he reproaches himself, vilifies himself and expects to be cast out and punished.” Freud (1917) qualified this distortion as “*a delusion of (mainly moral) inferiority*” (*p. 245*). In Margo et al.’s (1993) study on defensive styles, depression severity was associated with the amount of negatively biased self-perception in depressed inpatients. As shown in the following example, a patient used distortion in a similar way when she considered herself a “*crazy depressive*.” The patient started the first session of therapy saying she was an illegal immigrant and condemned herself as guilty of her brother’s suicide. Later in the session, she thought of herself as a murderer: “*I read books where someone killed someone*…. *These are the books I am interested in*…. *It gives me ideas, I could plan a murder*.” The therapist voiced the anger present in the patient and the patient completed the sentence: “… *everything I try to do leads me there, too*…. *Indeed, I am crazy [mumbling]. I am a crazy depressive*.”

Our findings are likely to measure state changes in psychotic defenses in a way that is similar to depressive defenses throughout a brief and intensive psychoanalytic therapy during a particular phase of the overall depressive course, namely, a crisis leading to hospitalization. Trait changes are likely to need a longer course of therapy. However, our findings may indicate that a decrease in psychotic level can be a first therapeutic step; whether this step occurs by addressing individual defenses or by containing them is still unknown and remains an open research question.

These results must be interpreted in light of several potential limitations. The small sample size limits the statistical power to detect change, meaning that small or medium effects of the treatment response may have been missed. Only cases with available recordings of the two sessions were included. The possibility that this represents a selection bias, in particular because the management of the recording was left to the therapist, could not be ruled out. However, we verified that the cases selected for this study are no different from the other cases in the research.

The study is heterogeneous in multiple ways. Different subtypes of depression and comorbidity were included. At intake, MADRS depression severity scores varied from 17 to 49. Controlling the additional treatment received during the hospitalization was not possible. Therefore, other unmeasured variables or variables that could not be included in the model, due to the sample size, may moderate the link between defense and outcome.

The short duration of the treatment and follow-up for problems that tend to be chronic do not reveal whether patients developed sustained improvement. Outcome measurement tends to “evaluate a particular moment in time rather than an ongoing experience” (Bond and Perry, 2004, p. 1666). Further study should examine longer treatment to improve the understanding of the mediating role of defensive functioning and defense mechanisms in therapy response and remission. Finally, change in defensive functioning was evaluated by comparing ratings of only two sessions (the second and the penultimate). Previous research has shown that the rating of more sessions may give a more representative measure of defensive functioning, particularly in terms of a relatively stable trait (Perry, 2001).

The assessment of defenses was only done at the beginning and end of the brief psychodynamic psychotherapy during hospitalization. Assessing defenses after 12 months of follow-up, which would have required interviews, was not possible. Therefore, the extent to which defenses change in the long term is not known. The study only assessed whether early changes in defenses predicted symptomatic improvement in our sample a year later. We did not also have a measure of structural change at follow-up, which makes interpreting these results beyond depressive vulnerability impossible, in terms of structural personality functioning change.

Another limitation is that the study does not address causal relationships. The psychoanalytic theory is that beneficial changes in defensive functioning result in symptomatic improvement. However, changes in defensive functioning and improvement of symptoms might be the effects of some other sort of therapeutic process, such as increases in attachment security due to a good therapeutic relationship. Improvement in defensive functioning could be a function of a common factor (e.g., the therapeutic alliance) that appears to predict improvement in all psychotherapeutic approaches.

This study complements previous work on how defenses predict outcomes in depressive disorders by examining the clinical significance of the results and by including psychotic defenses. As expected, ODF and specific low- and high-adaptive levels of defense changed during short psychodynamic psychotherapy and predicted treatment response. We showed that the addition of psychotic defenses allows a better prediction of the treatment response. As Babl et al. (2019) showed, future research must measure defensive functioning and defense mechanisms longitudinally to disentangle within- and between-patient effects of defenses and to achieve unbiased estimates that are more robust.

## Data Availability Statement

The data analyzed in this study are subject to the following licenses/restrictions: Data are only available for the first author and the third author (the statistician). Requests to access these datasets should be directed to yves.deroten@chuv.ch.

## Ethics Statement

The studies involving human participants were reviewed and approved by University of Lausanne Ethical Committee (April 12, 2010). The patients/participants provided their written informed consent to participate in this study.

## Author Contributions

YR designed the study, collected and coded the data, interpreted the results, and wrote the manuscript. SD coded the data and wrote the manuscript. FC did the statistical analysis and supported the interpretation of the results. J-ND designed the study and supported the interpretation of the results. GA designed the study, collected the data, and wrote the manuscript. All authors contributed to the article and approved the submitted version.

## Conflict of Interest

The authors declare that the research was conducted in the absence of any commercial or financial relationships that could be construed as a potential conflict of interest.
