# Supplementary material for: Defense Mechanisms and Treatment Response in Depressed Inpatients
Source: Front Psychol. 2021 Mar 18;12:633939. doi: 10.3389/fpsyg.2021.633939 (PMC8012720; doi:10.3389/fpsyg.2021.633939)
Supplement: Supplementary file 1 [file Table_1.DOCX]

Supplementary Table

**Table 1.** *Comparison Between Full Sample and Subsample on Demographic and Clinical Characteristics*

| Variable | Full sample  (N = 149) | Subsample  (*n* = 41) | Subsample vs reminders  *p* |
| --- | --- | --- | --- |
| Age | 43.1 (10.9) | 44.2 (9.6) | .661 |
| Gender (female) | 108 (72.5%) | 27 (65.8%) | .121 |
| Education (years) | 10.6 (3.0) | 10.1 (2.9) | .414 |
| Marital status |  |  |  |
| Single | 34 (26.8%) | 7 (17.1%) |  |
| Couple | 41 (32.3%) | 18 (43.9%) |  |
| Divorced/widowed | 52 (40.9%) | 16 (39.0%) | .211 |
| Chronicity | 66 (44.3%) | 21 (51.2%) | .267 |
| Tentamen | 66 (51.2%) | 19 (46.3%) | .704 |
| Early onset | 41 (32.3%) | 13 (31.7%) | .680 |
| Duration of current episode | 65.7 (98.4) | 77.2 (100.5) | .507 |
| Childhood trauma (CTQ) | 2.2 (0.85) | 2.3 (0.82) | .551 |
| Length of hospital stay | 36.6 (24.5) | 44.0 (40.3) | .234 |
| Depression (MADRS) | 30.1 (6.7) | 31.2 (6.6) | .187 |

*Note*. Statistical tests were *t*-test or Fisher exact test; CTQ = childhood trauma questionnaire; MADRS = Montgomery-Asberg Depression Rating Scale
