# Supplementary material for: Defense Mechanisms and Treatment Response in Depressed Inpatients
Source: Front Psychol. 2021 Mar 18;12:633939. doi: 10.3389/fpsyg.2021.633939 (PMC8012720; doi:10.3389/fpsyg.2021.633939)
Supplement: Supplementary file 2 [file Table_2.DOCX]

Supplementary Table

**Table 2.** *Relation Between Defenses and Treatment Response and Remission Measured by the QIDS-SR_16_*

|  | Response | | | | |  | | Remission | | | | |  |
| --- | --- | --- | --- | --- | --- | --- | --- | --- | --- | --- | --- | --- | --- |
| Defenses | Estimate | *p* | | 95% CI | | | Estimate | | *p* | 95% CI | | |  |
|  |  | |  | LL | UL | |  | |  | | LL | UL |  |
| **ODF** |  |  | |  |  | |  | |  | |  |  |  |
| Time | -0.515 | .000 | | -0.709 | -0.322 | | -0.470 | | .002 | | -0.760 | -0.179 |  |
| Response | -0.511 | .008 | | -0.879 | -0.142 | | -0.268 | | .234 | | -0.714 | 0.179 |  |
| Time x Response | 0.421 | .003 | | 0.157 | 0.686 | | 0.238 | | .157 | | -0.095 | 0.572 |  |
| **Mature Category/High adaptive Level** | | | | | | | | | | | | |  |
| Time | -6.311 | .000 | | -8.997 | -3.624 | | -4.620 | | .026 | | -8.667 | -0.572 |  |
| Response | -2.167 | .163 | | -5.230 | 0.896 | | 2.075 | | .251 | | -1.496 | 5.645 |  |
| Time x Response | 5.467 | .005 | | 1.798 | 9.132 | | 1.643 | | .480 | | -3.013 | 6.298 |  |
| **Obsessional Level** | | | | | | | | | | | | |  |
| Time | -5.226 | .014 | | -9.333 | -1.120 | | -5.980 | | .040 | | -11.660 | -2.997 |  |
| Response | -7.836 | .027 | | -14.746 | -0.925 | | -9.471 | | .021 | | -17.481 | -1.461 |  |
| Time x Response | 3.208 | .254 | | -2.398 | 8.814 | | 3.274 | | .317 | | -3.259 | 9.806 |  |
| **Action Level** | | | | | | | | | | | | | |
| Time | 2.011 | .261 | | -1.557 | 5.578 | | 1.290 | | .600 | | -3.650 | 6.230 |  |
| Response | 6.788 | .045 | | 0.028 | 7.538 | | 2.706 | | .244 | | -1.888 | 7.300 |  |
| Time x Response | -1.551 | .523 | | -6.421 | 3.318 | | -0.148 | | .958 | | -5.829 | 5.533 |  |
| **Psychotic Level** | | | | | | | | | | | | | |
| Time | 1.216 | .377 | | -1.538 | 3.970 | | 1.860 | | .331 | | -1.964 | 5.684 |  |
| Response | 4.194 | .022 | | 0.626 | 7.762 | | 2.913 | | .178 | | -1.357 | 7.183 |  |
| Time x Response | -2.920 | .124 | | -6.680 | 0.839 | | -2.925 | | .186 | | -7.322 | 1.473 |  |
| **Immature Category** | | | | | | | | | | | | | |
| Time | 9.347 | .000 | | 5.117 | 13.578 | | 9.570 | | .003 | | 3.496 | 15.644 |  |
| Response | 10.741 | .006 | | 3.178 | 18.304 | | 6.306 | | .175 | | -2.903 | 15.515 |  |
| Time x Response | -7.197 | .016 | | -12.972 | -1.422 | | -5.402 | | .126 | | -12.388 | 1.583 |  |

*Note*. Only Level/Category with significant results for Response or Time x Response are displayed. Immature defenses include psychotic, action, borderline and disavowal levels; ODF = Overall defensive functioning; CI = confidence interval; LL = lower limit; UL = upper limit.
